# Vacuum-Implemented Removal of Lead Vegetations in Cardiac Device-Related Infective Endocarditis

**DOI:** 10.3390/jcm11154600

**Published:** 2022-08-07

**Authors:** Vincenzo Tarzia, Matteo Ponzoni, Giuseppe Evangelista, Chiara Tessari, Emanuele Bertaglia, Manuel De Lazzari, Fabio Zanella, Demetrio Pittarello, Federico Migliore, Gino Gerosa

**Affiliations:** 1 Cardiac Surgery Unit, Department of Cardiac, Thoracic, Vascular Sciences and Public Health, University of Padua, 35128 Padua, Italy; 2 Cardiology Unit, Department of Cardiac, Thoracic, Vascular Sciences, and Public Health, University of Padua, 35128 Padua, Italy; 3 Anesthesia and Intensive Care Unit, University of Padua, 35128 Padua, Italy

**Keywords:** AngioVac, lead, pacemaker, CDRIE

## Abstract

When approaching infected lead removal in cardiac device-related infective endocarditis (CDRIE), a surgical consideration for large (>20 mm) vegetations is recommended. We report our experience with the removal of large CDRIE vegetations using the AngioVac system, as an alternative to conventional surgery. We retrospectively reviewed all infected lead extractions performed with a prior debulking using the AngioVac system, between October 2016 and April 2022 at our institution. A total of 13 patients presented a mean of 2(1) infected leads after a mean of 5.7(5.7) years from implantation (seven implantable cardioverter-defibrillators, four cardiac resynchronization therapy-defibrillators, and two pacemakers). The AngioVac system was used as a venous–venous bypass in six cases (46.2%), venous–venous ECMO-like circuit (with an oxygenator) in five (38.5%), and venous–arterial ECMO-like circuit in two cases (15.4%). Successful (>70%) aspiration of the vegetations was achieved in 12 patients (92.3%) and an intraoperative complication (cardiac perforation) only occurred in 1 case (7.7%). Subsequent lead extraction was successful in all cases, either manually (38.5%) or using mechanical tools (61.5%). The AngioVac system is a promising effective and safe option for large vegetation debulking in CDRIE. Planning the extracorporeal circuit design may represent the optimal strategy to enhance the tolerability of the procedure and minimize adverse events.

## 1. Introduction

Trends in cardiac device implantation in recent decades have reflected the global population aging phenomena [1,2]. With growing large-scale adoption of transcatheter aortic valve replacement and the related risk of atrio-ventricular block, the implementation of guidelines for primary and secondary prevention of cardiac adverse events in heart failure, as well as the intrinsic senescence of conduction tissues in older individuals, the use of pacemakers (PM) and implantable cardioverter-defibrillators (ICD) has become a routine practice.

Late septuagenarians and octogenarians represent the most frequently treated age group [1,2], where multi-organ comorbidities are extremely common. In this scenario, the annual incidence of cardiac device-related infective endocarditis (CDRIE) ranges from 1.5% to 2.5%, generating a significant social and mortality burden [1,3,4]. According to the most recent European Heart Rhythm Association (EHRA) guidelines [5], infected lead extraction is always recommended and a percutaneous approach is the first choice (TLE: transvenous lead extraction). Although the high rates of successful percutaneous removal, in-hospital mortality can reach 2.3%, especially when a systemic infection is suspected [6]. In these settings, as well as in large (>20 mm) vegetations, a vacuum-assisted aspiration of the mass or a conventional surgical approach is suggested [5].

The AngioVac system (AngioDynamics, Latham, NY, USA) is a novel percutaneous technology that includes an aspiration cannula combined with a filter, a centrifugal pump, and a venous or arterial reinfusion cannula, intended for the intravascular aspiration of right-sided masses. This device has found an application for CDRIE vegetations debulking before lead extraction [7,8,9,10,11,12,13,14]. A systematic review of literature by Rusia et al. in 2019 collected a total of 88 patients in whom this device has been successfully adopted to treat CDRIE in 97.7% of cases [15]. However, the level of evidence is still low [5] and the feasibility and efficacy profiles of the AngioVac system are still under active investigation [16]. Moreover, the extracorporeal circuit′s configurations that can be adopted with this device have not been investigated systematically.

We report our single-center experience with the percutaneous vacuum-assisted removal of large CDRIE vegetations using the AngioVac system before transvenous lead extraction. In particular, we provide a structured decisional algorithm for the planning of the extracorporeal circuit’s configurations to provide the most adequate respiratory and/or hemodynamic support during the procedure.

## 2. Materials and Methods

### 2.1. Study Population

We conducted a retrospective review of all consecutive infected lead extractions, performed with a prior debulking of vegetations using the AngioVac system, between October 2016 and April 2022 at our institution. All patients signed the informed consent to the operation and use of data for scientific purposes. The study was approved by the local ethics committee (protocol 39677, June 2022).

Baseline clinical and demographic characteristics of patients, intraoperative variables, and in-hospital events were collected by hospital charts and imaging data review. Successful mass removal was defined by the aspiration of >70% of the mass as displayed on intraoperative transesophageal echocardiography, and device safety by the rate of procedural complications, as previously described [13,16].

### 2.2. Procedure Planning

**Figure 1:**
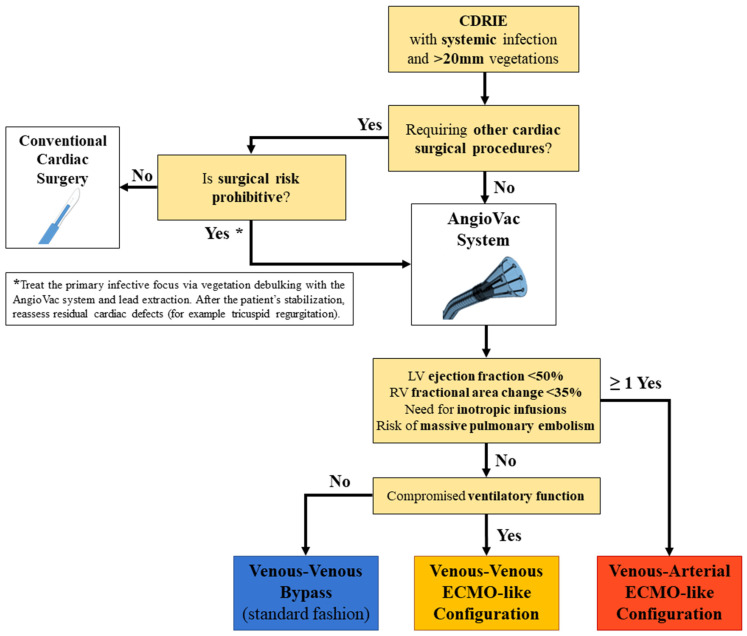
Decisional algorithm for management of CDRIE with large vegetations or systemic infection.

According to the guidelines [5], all patients with large (>20 mm) CDRIE vegetations or systemic infection underwent a multidisciplinary evaluation to plan a surgical extraction of the infected device [17] (Figure 1). When feasible, percutaneous vacuum-assisted aspiration of the mass with the AngioVac system was the preferred approach, followed by a transvenous lead removal.

**Figure 2:**
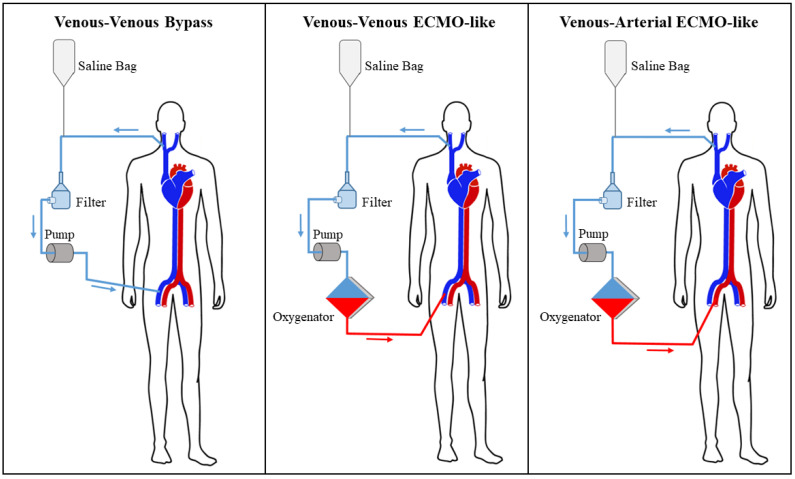
Different configurations of the AngioVac system circuit.

The respiratory and hemodynamic status of the patient guided the subsequent decisional steps. Three different configurations of the extracorporeal circuit were adopted at our institution (Figure 2). If the patient was hemodynamically stable and the cardiac function was preserved, a standard venous–venous bypass circuit where the AngioVac cannula suctions venous blood which is reinfused through another venous vessel was used. This circuit was upgraded with an oxygenator, realizing a venous–venous ECMO-like configuration, if the patient had compromised ventilatory function that required respiratory support during the procedure. Finally, in the case of septic shock, impaired ventricular function, or larger masses with a consistent risk of hemodynamically impacting pulmonary embolization, a venous–arterial ECMO-like configuration was chosen. In this circuit design, the AngioVac aspiration cannula filters venous blood was returned to the patient through an arterial cannula (after being oxygenated), ensuring adequate circulatory support.

### 2.3. Operative Technique

The procedure was performed under general anesthesia and the operating room was equipped with three-dimensional transesophageal echocardiography and fluoroscopy. For pacemaker-dependent patients, a temporary transvenous pacing device was placed. Angiography of the left subclavian vein was performed and a stiff guidewire was placed from the LFV or RFV into the RIJV to potentially place an occlusion balloon in case of vessel perforation. The cardiac device pocket was incised and leads were prepared with a locking stylet (Liberator, Cook Vascular Inc., Bloomington, IN, USA) and a compression coil (One-Tie, Cook Vascular Inc.).

A 5000 IU bolus of unfractionated heparin was administered to achieve partial heparinization with a target activated clotting time >180 s. The 22F AngioVac aspiration cannula was placed in the right internal jugular vein (RIJV), the right or left femoral vein (RFV, LFV), or a combination of these accesses. To facilitate the insertion of the suction cannula, a 26 Fr Gore DrySeal venous sheath (W.L. Gore & Associates, Newark, DE, USA) was used. All the three generations of the AngioVac cannula were used: first generation with a straight tip and a balloon-activated funnel; second generation with a 20° angled tip and a balloon-activated funnel; and third generation with a 20° or 180° angled tip and a self-expanding nitinol funnel.

Subsequently, the reinfusion cannula was placed in another venous or arterial vessel, depending on the intended strategy. If a venous–venous configuration was planned, all vessels were cannulated percutaneously preferentially. In the case of a venous–arterial ECMO-like configuration, a surgical exposition of the femoral vessels was performed. Vessels’ cannulation and the extracorporeal circuit set-up was carried out by the cardiac surgeon, who was assisted by the electrophysiologist for lead, guide, and cannula visualization using fluoroscopy.

The AngioVac cannula was driven under transesophageal echocardiographic guidance into the right of the heart and used for the debulking of lead vegetations by the cardiac surgeon. Even if a successful (>70%) removal of the mass was achieved, it was common that small residual vegetations could persist onto the leads, due to strong adherences. To prevent the embolization of these residual masses, the AngioVac cannula was left in place in the right atrium close to the lead and the aspiration was continued while the extraction was accomplished. This allowed the suction of small vegetation debrides that were mobilized during the catheters′ extraction. TLE was performed by the electrophysiologist either manually or mechanically using Evolution RL Lead Extraction tools (Cook Vascular Inc.), depending on the encountered tissue resistance, as we previously described [17]. The AngioVac cannula was extracted and transesophageal echocardiography was used to rule out valvular injury and pericardial effusion. The DrySeal sheath and the reinfusion cannula were removed and hemostasis of access sites was completed. Finally, samples from the device pocket and leads were sent for microbiological analysis.

### 2.4. Statistical Analysis

Quantitative variables were summarized as mean (standard deviation (SD)) and median (interquartile range (IQR)) and categorical variables as counts and percentages. Analyses were performed using SPSS 23.0 (IBM Corporation, Armonk, NY, USA).

## 3. Results

During the study period, we performed 13 AngioVac-assisted infected lead removals (13 males; mean age 63.6 [12.2] years). Patients presented a mean of 2 (1) infected leads after a mean of 5.7 (5.7) years from implantation. In seven cases (53.8%), the infected device was an ICD, in four (30.8%) a cardiac resynchronization therapy-defibrillator (CRT-D), and in the other two (15.4%) a PM. Complete preoperative characteristics are summarized in Table 1. Of note, two patients (15.4%) had a previous cardiac surgery operation (coronary artery bypass graft surgery; correction of double-outlet right ventricle with ventricular septal defect closure and right ventricle to pulmonary artery homograft), five (38.5%) had moderate- or severe-associated tricuspid valve regurgitation due to the mass interference with leaflet coaptation, and eight (61.5%) presented positive blood cultures (see Table 2 for isolated germs).

**Figure 3:**
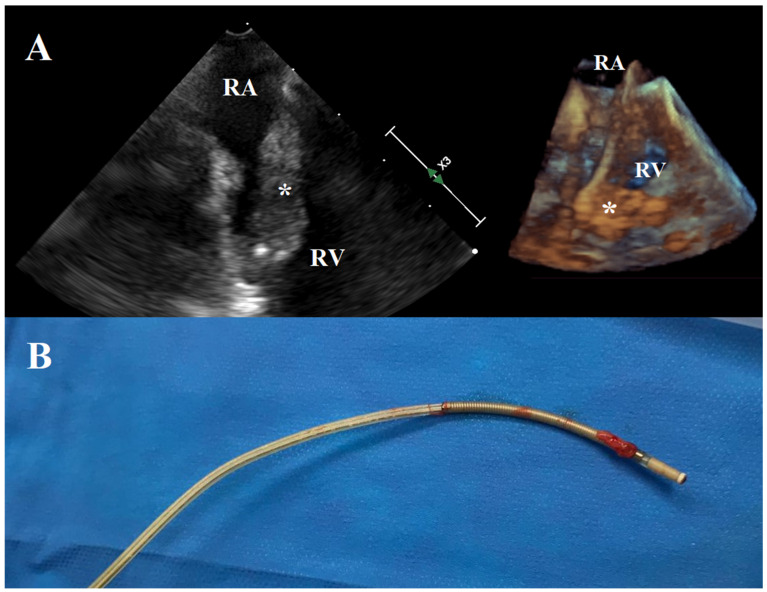
Intraoperative echocardiographic view of large lead vegetations (30 mm × 90 mm, Panel **A**). Extracted PM lead (Panel **B**). RA: right atrium. RV: right ventricle. *: vegetation.

In most of the cases (nine patients, 69.2%), the mass was single and its mean diameter was 37.2 (18.6) mm (Figure 3). Two patients (15.4%) had a mass < 25 mm, two (15.4%) between 25 and 30 mm, three (23.1%) between 30 and 40 mm, and six (46.2%) ≥ 40 mm. Mass debulking was conducted using the AngioVac system in three different configurations: venous–venous bypass circuit in six cases (46.2%), venous–venous ECMO-like circuit in five (38.5%) to provide respiratory support, and venous–arterial ECMO-like circuit in two (15.4%) to ensure full hemodynamic stabilization during the procedure. The cannulation sites for blood aspiration and reinfusion are described in Table 3. Subsequent extraction of infected leads was achieved manually in five cases (38.5%) and using dedicated mechanical tools (Evolution RL Lead Extraction, Cook Vascular Inc.) in 8 (61.5%).

Successful aspiration (>70%) of the mass was achieved in 12 patients (92.3%, Video S1). In particular, a complete (100%) aspiration of the mass was accomplished in all successful cases. In the other case, a correct alignment of the aspiration cannula (first generation with a straight tip) with the tricuspid valve was not possible. A partial (50%) aspiration of the vegetation was accomplished (residual mass <20 mm) and the infected lead was subsequently extracted without complications. In all cases, all the infected leads were successfully extracted.

We reported one intraoperative complication (7.7%): during the insertion of the DrySeal guide, cardiac perforation occurred at the right ventricular free wall, requiring conversion to sternotomy to control the bleeding. However, the mass was successfully removed using the AngioVac cannula placed directly into the right atrium, through the atrial appendage. Of note, mass fragmentation and/or its embolization during the procedure never occurred.

In the postoperative period, the most common morbidities were: acute kidney injury requiring renal replacement therapy in four (30.8%), new-onset atrial fibrillation in two (15.4%), access site injury in one (7.7%), severe tricuspid regurgitation requiring tricuspid valve replacement (although severe regurgitation was present preoperatively) in one (7.7%), and the need for temporary venous–arterial ECMO support in one (7.7%). Complete postoperative outcomes are presented in Table 4. The grade of tricuspid valve regurgitation at discharge improved or remained stable in all cases. Noticeably, thirty-day mortality was 0%.

## 4. Discussion

In the current era, the landscape of infective endocarditis has dramatically changed. Implanted cardiovascular devices have increased exponentially, as well as the clinical complexity of patients [1,18]. The presence of an intravascular foreign material is the most important predisposing factor for early or late microorganism colonization, an issue that can be only partially controlled by improved implantation techniques and device technologies. In fact, early CDRIE can affect more than 10% of patients, especially in the older age groups [19], and the risk of late CDRIE cumulates every year from implantation [1]. Prompting the removal of the infected device can impact the patient′s outcome positively [20], particularly when a systemic infection is present. However, large vegetations and septic status require further surgical consideration.

At our institution, the strict and valuable collaboration between electrophysiologists and cardiac surgeons has been translated into a multidisciplinary approach to complex CDRIE [17]. Given the promising efficacy and safety of the AngioVac system for the treatment of life-threatening intracardiac masses [16,21,22,23], we incorporated this device in our decisional algorithm of CDRIE (Figure 1), as a valid alternative to conventional surgery. In our experience, we performed a total of 13 percutaneous vacuum-assisted removals of large CDRIE vegetations using the AngioVac system, with encouraging results.

Our treated population was mainly composed of individuals who were implanted with ICD or CRT-D for primary or secondary prevention of cardiac adverse events. This aspect underlines the intrinsic fragility of our cohort of patients, in whom a previous cardiac arrest or chronic congestive heart failure affected more than half of the individuals (Table 1). In this setting, infected lead extraction and related risk of embolization can be poorly tolerated and may precipitate dramatically. Many authors attempted to quantify the risk of pulmonary embolization during these procedures but reported rates are sparse, ranging from 0 to 55% [24,25,26,27]. Different imaging techniques and thresholds for the definition of significant pulmonary embolization can partially account for this inhomogeneity of data. Moreover, a linear correlation between the risk of embolization and lead vegetation size has not been demonstrated [28,29]. Recently, Caiati et al. concluded that CDRIE vegetation size is not a determinant of postoperative outcomes of percutaneous lead extraction [29]. However, the maximal dimension of vegetations was below 10 mm in most of the patients in their analysis. Thus, these results can hardly be translated into our cohort, where vegetations measured up to 40 mm on average (Table 1). We speculate that the paradigm of vegetation size as a prognostic predictor in valvular infective endocarditis [30] can be applied to our treated population, in whom a surgical consideration before extraction was mandatory to guarantee a safe lead extraction.

Although a clear benefit of vacuum-assisted debulking of lead vegetations over a conventional percutaneous extraction has not yet been proven [9], the preliminary results of the AngioVac system for the aspiration of intracardiac masses are promising [16]. Successful removal rates vary depending on the specific origin and location of the mass, ranging between 60 and 80% in larger registries and series [16,31]. However, when considering CDRIE vegetations debulking, procedural success is >90% in most of published reports [7,11,12,13,32]. In a recent multi-center cohort including 101 patients, Starck et al. proved a percutaneous vacuum-assisted aspiration to be completely successful in 94% of CDRIE [13], similarly to conventional TLE procedures [33]. Our work aligns with these outstanding results, with 12/13 successful procedures (92.3%). The only patient in whom a partial debulking (50%) was performed due to an unsatisfactory alignment of the first-generation aspiration cannula (with a straight tip) with the tricuspid valve could now benefit from the most recent cannula’s designs. In fact, the third-generation AngioVac cannulas can mount 20° and 180° angled tips, which further enhance the mobility and adaptability of the system (Video S1). Moreover, we suggest that a dual approach using two different aspiration accesses (RFV + RIJV) might allow a complete suction of the vegetation, particularly when the femoral approach does not consent a satisfactory targeting of the mass. In our experience, this strategy permitted a successful mass debulking in one patient, when the third-generation cannulas were not available.

Intraoperative complications commonly related to the AngioVac procedure entail cardiac perforation, valve damage, arrhythmias, access sites injury, and mass fragmentation with subsequent embolization [16,31]. As previously stated, these events could rapidly lead to circulatory collapse in fragile patients. Moreover, CDRIE could be associated with septic status, a condition in which a venous–venous bypass could be poorly tolerated [13]. The Indigo Thrombectomy system (Penumbra Inc, Alameda, CA, USA) is an alternative percutaneous cannula that has been adopted to suction lead vegetations in hemodynamically unstable patients because it does not require an extracorporeal venous–venous bypass [34]. However, the inability to return blood to the patient generates considerable blood loss during the procedure [34].

To overcome all these issues, we opted for the AngioVac system and we tailored the extracorporeal circuit configuration to the patient′s preoperative hemodynamic status, cardiac performance, and the characteristics of the vegetations (Figure 1). Thanks to the exceptional versatility of the AngioVac system, the extracorporeal circuit can be adapted to the patient’s needs simply by moving the reinfusion cannula from a venous to an arterial vessel and interposing an oxygenator. In relatively low-risk patients, with preserved respiratory and cardiac function and affected by smaller vegetations, we routinely adopted the standard venous–venous bypass (six cases, 46.2%). In case of respiratory compromise of any kind, we included an oxygenator in the circuit (five cases, 38.5%), realizing a venous–venous ECMO-like configuration. This design guarantees that oxygenated blood is returned to the patient, supporting his/her ventilatory function that could be further impaired by fluid administration and hemodilution during the procedure. Finally, when cardiac performance is affected, in the case of septic status, or if extremely mobile and large vegetations are at high risk of massive pulmonary embolization, we chose a venous–arterial ECMO-like configuration (two patients, 15.4%) that can provide full hemodynamic support. Although an arterial reinfusion has rarely been adopted in larger cohorts treated with the AngioVac system (3.8% of cases in the RAPID registry [16]), we consider this approach a valid option to treat patients with prohibitive surgical risk safely, bypassing the well-known limitations of the standard venous–venous circuit. In the multi-center experience reported by Starck et al. (in which a venous–venous configuration was used in all cases), two of the three major complications that occurred during the CDRIE vegetation debulking were hemodynamic collapses of patients [13]. We speculate that planning a venous–arterial ECMO-like configuration in high-risk patients has the potential to further minimize this complication, guaranteeing adequate circulatory support during the aspiration maneuvers.

With this strategy, we reported a very low intraoperative complication rate (7.7%), which is consistent with the previous series [11,12,13,15,32]. Similarly, 30-day mortality was absent and postoperative complications directly device-related affected only a small percentage of patients (Table 4). Particular consideration during lead extraction should be given to preserving the tricuspid valve competence, to avoid the need for a subsequent reparative open surgery. George et al. observed a considerable rate (43.5%) of worsening regarding the severity of regurgitation after the procedure [32], which can be related to both the initial vacuum-assisted vegetation debulking and the lead extraction per se. In our experience, tricuspid regurgitation remained stable or slightly improved in all cases, underlying the presence of both a mechanism of mass interference with leaflet coaptation and an intrinsic valve disease (mainly due to annular dilatation and leaflet damage by leads and CDRIE). In only one case, tricuspid regurgitation remained severe after infected lead extraction, requiring surgical replacement. We herein support the safety of the AngioVac device in also avoiding tricuspid valve damage during mass debulking in CDRIE.

## 5. Limitations

The main limitations of our work are the small number of treated patients, its retrospective design, and the lack of a control population, although larger single-center experiences with the AngioVac system in CDRIE are rare. A specific sub-analysis of the ongoing prospective RAPID registry [16] is hoped to give better insight into the role of vacuum-assisted debulking of vegetations before lead extraction. Finally, further data are needed to prove the efficacy and safety of the venous–arterial ECMO-like configuration of the AngioVac system on a larger scale.

## 6. Conclusions

The AngioVac system represents a promising effective option for large vegetation debulking before infected lead extraction in CDRIE. The extracorporeal circuit design can be tailored to the patient’s hemodynamic status, cardiac performance, and vegetations characteristics. With this strategy, we reported an excellent successful removal rate, with a concomitant acceptable intraoperative complication rate. A percutaneous vacuum-assisted infected lead extraction emblematizes the valuable collaboration between electrophysiologists and cardiac surgeons in managing high-risk CDRIE.

## Figures and Tables

**Table 1:** Preoperative characteristics of patients (n = 13).

	Overall (n = 13)	
	Mean (SD)	Median (IQR)
Age (years)	63.6 (12.2)	67.1 (55.8–73.6)
Weight (kg)	73.9 (10.2)	76 (66.5–81)
Height (m)	1.73 (0.06)	1.72 (1.70–1.76)
Body surface area (m²)	1.87 (0.13)	1.88 (1.77–1.97)
Body mass index (kg/m²)	24.6 (3.4)	25.1 (21.1–26.3)
Serum creatinine (mg/dL)	1.12 (0.88)	0.87 (0.83–1)
Hemoglobin (g/L)	11.1 (1.9)	10.2 (9.7–12.7)
Left ventricular ejection fraction	0.43 (0.13)	0.36 (0.35–0.54)
Right ventricular fractional area change	0.42 (0.11)	0.41 (0.33–0.52)
Time from device implantation (years)	5.7 (5.7)	5 (0.9–9.4)
Number of infected leads	2 (1)	2 (1–3)
Mass dimension (mm)	37.2 (18.6)	39 (26–40.5)
MELD score	11 (5)	9 (8–12)
Karnofsky scale	71 (20)	70 (60–90)
Zubrod scale	2 (1)	2 (1–3)
	**N**	**%**
Male	13	100
Comorbidities		
Dyslipidemia	8	61.5
Arterial hypertension	6	46.2
Previous cardiac arrest	5	38.5
Smoke	4	30.8
Diabetes mellitus	4	30.8
Congestive heart failure	2	15.4
Renal replacement therapy	1	7.7
Previous cerebrovascular accident	1	7.7
Coronary artery disease	1	7.7
Previous cardiac surgery	2	15.4
Previous percutaneous coronary intervention	4	30.8
Preoperative anticoagulation	6	46.2
Preoperative antiplatelet therapy	5	38.5
Positive blood culture	8	61.5
Preoperative inotropic support	1	7.7
Preoperative tricuspid valve regurgitation		
Absent	3	23.1
Mild	5	38.5
Moderate	3	23.1
Severe	2	15.4
Type of implanted device		
Implantable cardioverter-defibrillator	7	53.8
CRT-D	4	30.8
Pacemaker	2	15.2
Indication to implantation		
Secondary prevention of cardiac arrest	6	46.2
Primary prevention of cardiac arrest	5	38.5
Symptomatic atrio-ventricular block	2	15.2
Number of masses		
One mass	9	69.2
Two masses	3	23.1
Three masses	1	7.7

CRT-D: Cardiac resynchronization therapy-defibrillator.

**Table 2:** Isolated germs from preoperative blood cultures (n = 8) and from intraoperative samples (n = 3).

Isolated Germs from Preoperative Blood Cultures	Patients with Positive Blood Cultures (n = 8)	
	**N**	**%**
Staphylococcus epidermidis	2	25
Enterobacter cloacae	2	25
Enterobacter faecalis	1	12.5
Acinetobacter baumanii	1	12.5
Staphylococcus aureus	1	12.5
Staphylococcus lugdunensis	1	12.5
**Isolated Germs from Intraoperative Samples**	**Patients with Positive Intraoperative Samples (n = 3)**	
	**N**	**%**
Staphylococcus epidermidis	1	33.3
Staphylococcus warneri	1	33.3
Rhizobium radiobacter	1	33.3

**Table 3:** Intraoperative details and circuit configurations (n = 13).

	Overall (n = 13)	
	Mean (SD)	Median (IQR)
Total operative time (min)	237 (91)	230 (173–328)
Fluoroscopy time (min)	7.8 (5.8)	7.1 (2.7–13.7)
AngioVac time: cannulation, aspiration, decannulation (min)	95 (25)	90 (65–120)
	**N**	**%**
Cannula generation		
First generation	3	23.1
Second generation	5	38.5
Third generation	5	38.5
Venous–venous bypass configuration	6	46.2
Aspiration cannula site		
RFV	3	50
RIJV	2	33.3
RFV + RIJV	1	16.7
Reinfusion cannula site		
LFV	4	66.7
RFV	2	33.3
Venous–venous ECMO-like configuration	5	38.5
Aspiration cannula site		
RFV	2	40
RIJV	1	20
LFV	1	20
Right atrium	1	20
Reinfusion cannula site		
LFV	4	80
RFV	1	20
Venous–arterial ECMO-like configuration	2	15.4
Aspiration cannula site		
RFV	2	100
Reinfusion cannula site		
RFA	2	100
Type of lead removal		
Mechanical removal	8	61.5
Manual removal	5	38.5
Histopathological analysis		
Thrombus	13	100
Successful aspiration	12	92.3

LFV: left femoral vein. RIJV: right internal jugular vein. RFA: right femoral artery. RFV: Right femoral vein.

**Table 4:** Postoperative outcomes (n = 13).

	Procedure Survivors (n = 13)	
	Mean (SD)	Median (IQR)
Creatinine peak (mg/dL)	1.23 (0.56)	1.02 (0.83–1.62)
Hemoglobin nadir (g/L)	10.8 (1.3)	11.1 (9.5–11.6)
Left ventricular ejection fraction	0.44 (0.11)	0.40 (0.36–0.55)
Right ventricular fractional area change	0.38 (0.07)	0.40 (0.34–0.44)
Intensive care unit stay (days)	5 (8)	3 (1–4)
Total hospital stay (days)	26 (21)	21 (16–30)
Karnofsky scale at discharge	75 (31)	90 (70–95)
Zubrod scale at discharge	2 (1)	1 (1–3)
	**N**	**%**
Acute kidney injury	4	30.8
Renal replacement therapy	4	30.8
New-onset atrial fibrillation	2	15.4
Access site injury	1	7.7
Tricuspid valve replacement	1	7.7
Postoperative ECMO support	1	7.7
Tricuspid valve regurgitation at discharge		
Absent	4	30.8
Mild	5	38.5
Moderate	3	23.1
Severe	1	7.7
30-day mortality	0	0

## Data Availability

Data available on request to the corresponding author.

## Supplementary Materials

The following supporting information can be downloaded at: https://www.mdpi.com/article/10.3390/jcm11154600/s1, Video S1. A case of CDRIE treated with the AngioVac system.

## Abbreviations

CDRIE	cardiac device-related infective endocarditis
CRT-D	cardiac resynchronization therapy-defibrillator
ECMO	extracorporeal membrane oxygenation
ICD	implantable cardioverter-defibrillator
IQR	interquartile range
LFV	left femoral vein
PM	pacemaker
RFV	right femoral vein
RIJV	right internal jugular vein
SD	standard deviation
